# Alterations in *Per2*, *Bcl2* gene expression, and oxidative status in aged rats liver after light pulse at night

**DOI:** 10.1007/s41105-023-00495-9

**Published:** 2023-11-05

**Authors:** Rehab E. El-Hennamy, Heba A. Elmasry

**Affiliations:** Zoology and Entomology Department, Faculty of Science, Helwan University, Cairo, Egypt

**Keywords:** Light entrainment, Circadian rhythm, Clock genes, Oxidative stress, Apoptosis

## Abstract

The aging process is characterized by circadian rhythm disruption, in physiology and behavior, which could result from weak entrainment. Light is the most potent cue that entrains the central circadian clock, which in turn synchronizes peripheral clocks in animal tissues. Period 2 *(Per2*) is one of the clock genes that respond to light. Moreover, oxidative stress could entrain the clock. Therefore, the present work aimed to investigate the role of light when applied late at night on the *Per2*, B cell lymphoma 2 (*Bcl2*) gene expression, and oxidative status in aged rats. Aged rats were divided into a control group and a group exposed to a 30-min light pulse applied daily during the subjective night at 5 am (ZT 22) for 4 weeks. *Per2* and *Bcl2* gene expression were quantified in liver tissue. To evaluate oxidative status, Glutathione (GSH), nitric oxide (NO), and malondialdehyde (MDA) were estimated. The light pulse reduced the expression levels of *Per2* and *Bcl2* mRNA. Although it diminished the levels of malondialdehyde (MDA), nitric oxide (NO) levels were elevated and the glutathione (GSH) levels were declined. In conclusion, the light pulse late at night abolished *Per2* mRNA circadian rhythm and reduced its expression in the liver of the aged rat. Similarly, it diminished the anti-apoptotic gene expression, *Bcl2*. Moreover, it might attenuate oxidative stress through the reduction in MDA levels.

## Introduction

Circadian rhythm orchestrates many features of physiology and behavior, affecting hormonal rhythms, metabolism, locomotor activity, sleep/wake cycles, and cognitive performance [1]. It originates from a master clock in the suprachiasmatic nuclei (SCN) of the hypothalamus [2]. The molecular mechanism responsible for generating intracellular circadian rhythms is a transcriptional-translational feedback loop consisting of positive and negative limbs [3]. The positive loop comprises of CLOCK and BMAL1 proteins, that initiate the transcription of *Per1-2* and *Cry1-2* genes. The negative limb consists of PER and CRY proteins which translocate back into the nucleus and bind with the CLOCK/BMAL1 complex to inhibit their transcription. After 24 h, proteins are broken down, resulting in the re-activation of transcription by CLOCK/BMAL1 [4].

In mammals, the master SCN clock is entrained to the outer environment by time cues (zeitgeber). The most important time cue is light [5]. The SCN endogenous rhythms is adjusted to the environment by light received from the retina via the retinohypothalamic tract [6]. The photic stimuli generate a pathway in the SCN that end with the initiation of *Per1* and *Per2* transcription via the cAMP response element binding protein. These events cause an advance or delay to the clock gene's circadian rhythm in SCN [7].

The liver, the essential metabolic organ in the body, is synchronized mainly by the SCN clock through the polysynaptic autonomic neural pathways [8]. Other evidence has shown that peripheral clocks are also regulated by light and hormones independent of the SCN. Besides, the liver clock is entrained by other factors, such as feeding cues, glucocorticoids, insulin, and ghrelin [9].

The aging process is mainly associated with circadian rhythm disruption that is characterized by variations in the physiological state and behavior of many species. Rhythm disturbances include weak coupling with environmental rhythms and alterations in the daily rhythm of hormone secretion. Age-related disruption of circadian rhythms may be due to neurobiological changes in the SCN that includes a diminish in the number of its cells and its volume [10].

With proceeding in age, the uncontrolled cellular metabolism leads to a rise in free radicals production and hence the elevation of reactive oxygen species (ROS) that overwhelm the antioxidant scavenging response of the cells resulting in impaired homeostasis [11]. Consequently, elderly people are more vulnerable to infections and disabilities. To obtain a healthy aging and long life span, the ROS levels should be decreased. Previous studies on longevity, such as caloric restriction, stimulated basal metabolic rate and led to the accumulation of ROS and increased oxidative stress [12]. Therefore, new avenues are required to overcome the rise of ROS during aging and enhance well-being. In previous study, we found a fundamental role of light exposure during the night, at 5 am (ZT 22), to attenuate neurodegenerative signs accompanying the aging process [13]. Herein, we wanted to investigate whether light, as an entraining signal for the circadian clock, could induce Per2 gene expression and reduce oxidative stress in aged rat liver when applied at late night (ZT22).

## Material and methods

### Animals

The study was performed on aged male rats (*Rattus norvegicus*) 18–24 months old, weighing 340–360 g. Sixty rats were purchased from the breeding unit of the Animal House of the National Research Center (Giza, Egypt). They were kept to acclimatize under moderate temperatures 24 ºC and 12:12 light–dark cycle (LD 12:12) with free access to water and chewing rodent food for 1 week. Rats were handled under the standards and ethics of laboratory animal care at the zoology department, faculty of science, Helwan University. Approval No. HU-IACUC/Z/RE2408-33.

### Experimental protocol

Rats were divided into two groups. The aged control group (*n = *30), was maintained under LD 12:12 with light onset at 7:00 am (ZT 0) and offset at 19:00 pm (ZT12). The aged light pulse group (*n = *30) was maintained under LD 12:12 and subjected to artificial normal light (100 lx) at late night for 30 min from 5:00am (ZT 22) to 5:30am then the light was switched off until the beginning of the subjected day at 7:00 am (ZT 0). ZT 22 was chosen for the timing of light pulse as it was proven to induce *Per2* mRNA in the SCN of adult grass rats [14]. Hence, the nocturnal light may exert its effect on the liver through both autonomic and hormonal pathways from the SCN [15].

The experiment lasted for a month then five rats from both groups were sacrificed at ZT 0, ZT 4, ZT 8, ZT 12, ZT16, and ZT20 during the 24 h cycle. The liver was removed for physiological and molecular investigations.

### RNA isolation and RT-PCR

RT-PCR (Real-time reverse transcription followed by polymerase chain reaction) was performed in GIS (Cairo, Egypt) company according to the method of Sládek et al. [16]. Total RNA was isolated from 20–50 mg of homogenized liver tissue. 1 μg of RNA was reverse transcribed to cDNA. The cDNA reaction was diluted and then amplified in a 20 μl PCR reaction containing commercial SYBR Green and Hot Start Taq polymerase mix (QuantiTect SYBR Green PCR kit) with specific primers for *Per2*, *Bcl2* or housekeeping gene (β-2 microglobulin). Primer sequences used were as follows: *Per2* (GenBank accession no. NM_031678) forward: 5ʹ-CACGCAACGGGGAGTACATCACAC-3′; reverse: 5′-CAAGGGGAGGCTGCGAACACAT-3′, *Bcl2* (GenBank accession no. NM_016993.1) forward: 5ʹ-CTGGTGGACAACATCGCTCTG-3′; reverse:  5ʹ-GGTCTGCTGACCTCACTTGTG-3ʹ, B-2-m (GenBank accession no. 012 12) forward: 5′-CGCTCGGTGACCGTGATCTTTCTG-3′; reverse: 5′-CTGAGGTGGGTGGAACTGAGACACG-3′. Real-time PCR reactions were performed on a Light Cycler system (PR0241400824, version PCR 2.3.2, RT 1.1.12.23) with the following thermo profile: initial denaturation at 95 °C for 15 min, 55 cycles with 15-s denaturation at 94 °C, 20-s annealing at 55–62 °C (primer specific temperature), and 10-s elongation at 72 °C. At the termination of each run, melting curve analysis was performed to ascertain the occurance of a single amplicon. The expression of *Per2* clock gene and *Bcl2* was normalized to the expression of β-2-microglobulin.

## Measurement of oxidative status

### Estimation of MDA level

Liver tissue was homogenized in 50 mmol of Tris–HCl buffer (pH 7.4). MDA, the end product of lipid peroxidation, the level was measured in the supernatant by the thiobarbituric acid reaction method [17].

### Estimation of nitrite/nitrate (NO) level

The principle for NO estimation is based on the conversion of nitrate to nitrite that is acidified and then react with Greiss reagents [sulfanilamide (1% in 5% H3PO4) to produce the diazonium ion then combines with naphthalene diamine dihydrochloride (0.1%) forming reddish purple azo dye]. The chromophoric azo dye resulting from the Greiss reaction was measured at 515 nm by spectrophotometer [18].

### Estimation of GSH

To estimate GSH, samples were deproteinized then Ellman’s reagent (5,5-dithiol-bis-(2-nitrobenzoic acid); DTNB) was added. The formed yellow chromagen was then measured by a spectrophotometer at 412 nm [19].

### Statistical analysis

The data obtained in the present study were represented as mean ± S.E and were analyzed using Graph pad prism version 8. Significant differences between the two groups were determined by 2-way ANOVA followed by Sidak's multiple comparison test. Differences between time points in the same group were represented as box and whisker plots and were analyzed by Tukey’s multiple comparison test. Results with *p < *0.05 were considered significant.

## Results

### Influence of light pulse on *Per2* mRNA expression

**Fig. 1:**
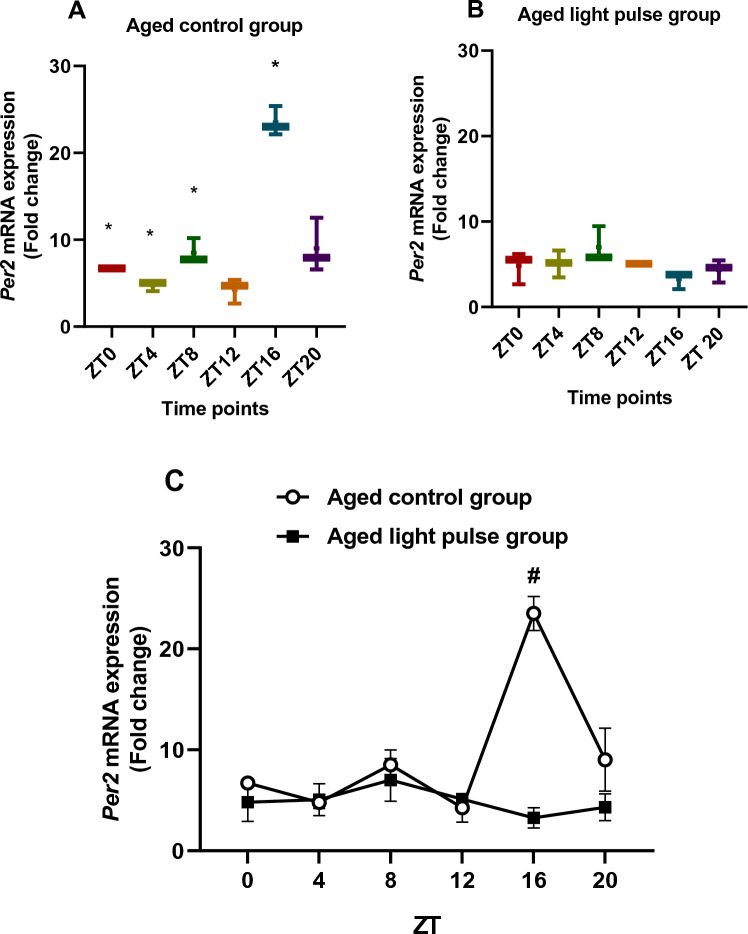
**A**, **B** Box and whisker plots showing the distribution of *Per2* mRNA levels in the liver tissue among different time points in aged control group (**A**) and aged light pulse group (**B**). * significance at ZT0, ZT4, and ZT8 vs ZT16. **C** A daily profile of *Per2* mRNA levels in the liver tissue of both aged control group and aged light pulse group. Data represented as mean ± SE. ^#^Significance at ZT16 between the 2 groups

In the aged control group (Fig. 1A**)**, box and whisker plots showed differences in the *Per2* mRNA expression during different time points because all boxes with median values did not overlap. The significant elevation (*p < *0.05) in this group was noticed at ZT16 compared to other time points (ZT0, ZT4, ZT8, ZT12) indicated by Tukey's multiple comparisons tests. These results showed a circadian rhythm of *Per2* mRNA in aged rat liver with the maximum value at early night. The aged group subjected to a light pulse at ZT22 (Fig. 1B) did not show any differences in *Per2* mRNA levels between time points except at ZT16 compared to ZT8 and ZT12, which was indicated by the box and whisker plots. These differences were not significant which means a loss of *Per2* mRNA rhythm after exposure to a 30 min light pulse. Two-way ANOVA showed a significant effect of time (*p = *0.0007), a significant effect of group (*p = *0.0002), and a significant interaction effect (*p < *0.0001) between the two groups*. Per2* mRNA expression was decreased significantly at ZT16 in the aged light pulse group compared to the aged control group, which was indicated by Sidak's multiple comparisons test (Fig. 1C). Thus, *Per2* mRNA circadian rhythm was repressed by light pulse exposure at late night.

### Influence of light pulse on *Bcl2* mRNA expression

**Fig. 2:**
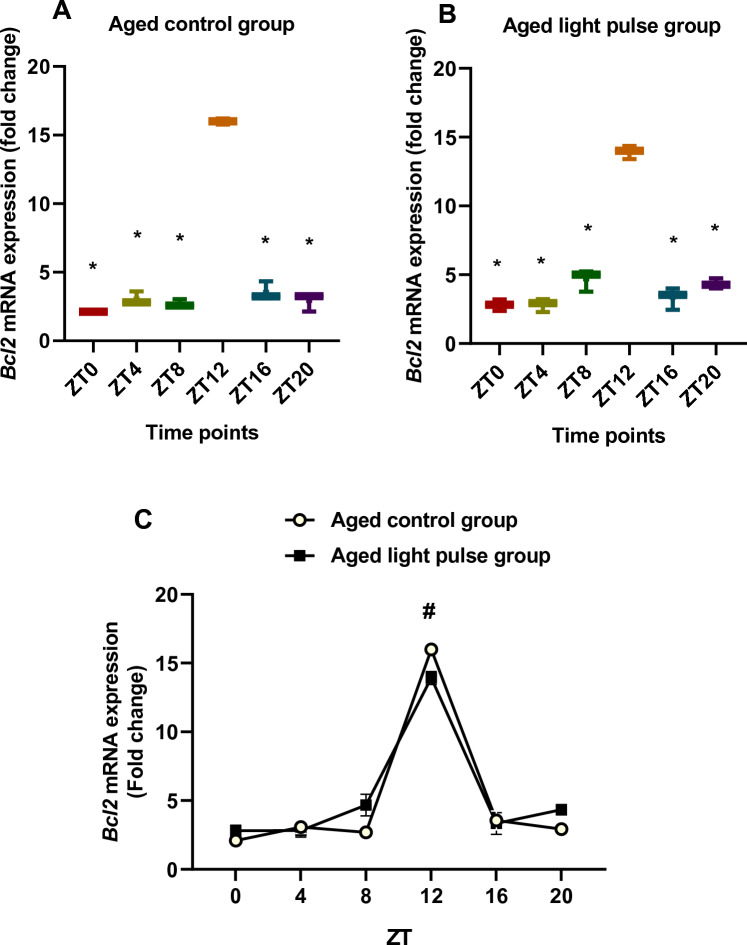
**A**, **B** Box and whisker plots showing the distribution of *Bcl2* mRNA levels in the liver tissue among different time points in aged control group (**A**) and aged light pulse group (**B**). * significance at ZT0, ZT4, ZT8, ZT16, ZT20 vs ZT12. **C** a daily profile of *Bcl2* mRNA levels in the liver tissue of both aged control group and aged light pulse group. Data represented as mean ± SE. ^#^ Significance at ZT12between the 2 groups

In aged rats, the *Bcl2* mRNA expression showed differences at ZT0 and ZT12 compared to other time points that appeared by box and whisker plots. The significant increase (*p < *0.05) was at ZT12 compared to other time points, ZT0, ZT4, ZT8, ZT16, and ZT20, (Fig. 2A). Similarly, rats exposed to a 30-min light pulse showed a significant increase (*p < *0.05) at ZT12 above other time points. But box and whisker plots showed another difference in *Bcl2* mRNA between ZT8 and other time points (Fig. 2B) as their boxes with median values did not overlap. Two-way ANOVA revealed a significant effect of time (*p < *0.0001). The effect of the group was not significant. The significant interaction (*p < *0.0001) between groups showed a significant difference between them at specific time points. *Bcl2* mRNA expression was reduced significantly (*p < *0.05) in the aged light pulse group compared to the control group at ZT12**.** These results clarify a circadian rhythm in *Bcl2* mRNA with a peak at ZT12 in both groups which was not affected by the light pulse (Fig. 2C).

## Effect of light pulse on oxidative status

### MDA levels

**Fig. 3:**
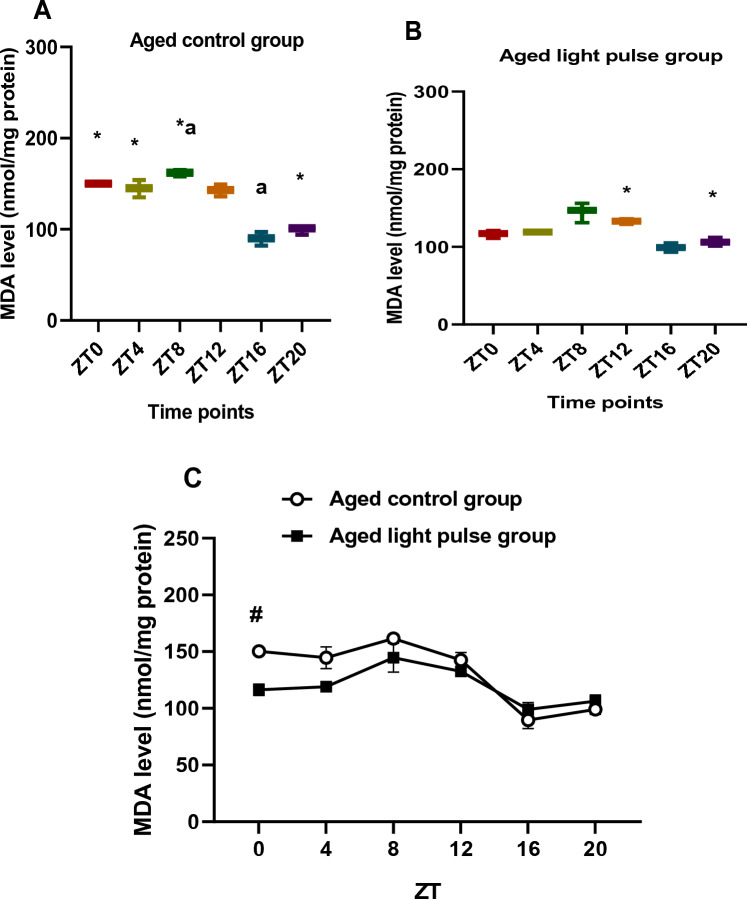
**A**, **B** Box and whisker plot showing the distribution of MDA levels in the liver tissue among different time points in aged control group (**A**) and aged light pulse group (**B**). In the aged control group (**A**), * significance at ZT0, ZT 4, ZT8 vs ZT 20; ^a^ significance at ZT8 vs ZT 16. In the aged light pulse group (**B**), *significance between ZT 12 and ZT20. **C** A daily profile of MDA levels in the liver tissue of both aged control group and aged light pulse group. Data represented as mean ± SE. ^#^ Significance at ZT0 between the 2 groups

MDA levels in the aged control group varied during different time points (ZT0, ZT4 vs ZT8, ZT16, ZT20 and ZT12 vs ZT8, ZT16, and ZT20) that appeared in the differences between boxes and median values of box and whisker plots**.** A significant elevation (*p < *0.05) was during the day at ZT0, ZT4, and ZT8 compared to ZT20, and at ZT8 compared to ZT16 (Fig. 3A). Variations in MDA levels, in the aged light pulse group, appeared during all time points except between ZT0 and ZT4 as shown by box and whisker plots. A significant increase in MDA levels was noticed at ZT12, at the beginning of the night, compared to ZT20 (Fig. 3B). Two-way ANOVA revealed a significant effect of time (*p < *0.0001), a significant effect of group (*p = *0.0006), and a significant interaction effect between groups (*p < *0.0001). A significant decline (*p < *0.05) in MDA levels was noticed in the aged light pulse group compared to that in the aged control group at ZT 0 as indicated by Sidak's multiple comparisons test (Fig. 3C).

### NO level

**Fig. 4:**
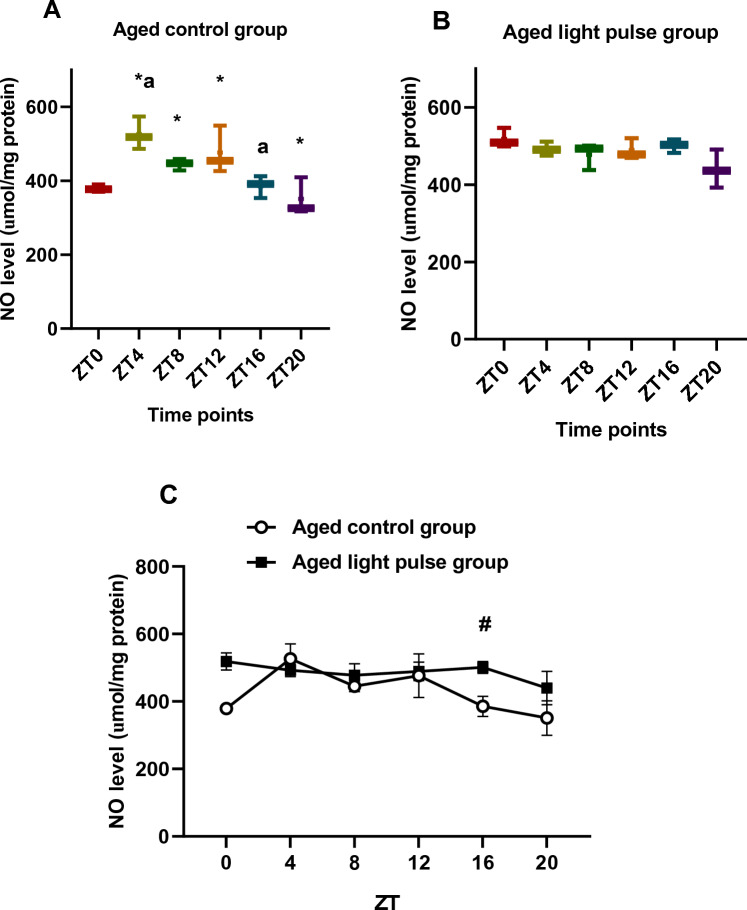
**A**, **B** Box and whisker plot showing the distribution of NO levels in the liver tissue among different time points in aged control group (**A**) and aged light pulse group (**B**). In the aged control group (**A**), * significance at ZT 4, ZT8, and ZT12 vs ZT 20; ^a^ significance at ZT4 vs ZT 16. In the aged light pulse group (**B**), no significant changes were observed. **C** A daily profile of NO levels in the liver tissue of both aged control group and aged light pulse group. Data represented as mean ± SE. ^#^ Significance at ZT16 between the 2 groups

Box and whisker plots showed differences in NO level during all time points except between ZT8 and ZT12, in the aged control rats. NO level showed the minimum value at ZT20, whereas the maximum significant values (*p < *0.05) were recorded during the afternoon and at the beginning of the dark phase at ZT4, ZT8, and ZT12. Additionally, the values of NO at ZT4 was significantly higher than that at ZT16 (Fig. 4A). In the aged-light pulse group, non-significant changes were detected in NO level during the different time points, but box and whisker plots showed differences in NO level during all-time points except at ZT4, ZT8, and ZT16 (Fig. 4B). Two-way ANOVA revealed a significant effect of time (*p < *0.0026), a significant effect of group (*p = *0.0007), and a significant interaction effect between groups (*p < *0.0001). NO levels were elevated significantly in the aged-light pulse group compared to the aged control one at ZT16, which was indicated by Sidak's multiple comparisons test (Fig. 4C).

### GSH levels

**Fig. 5:**
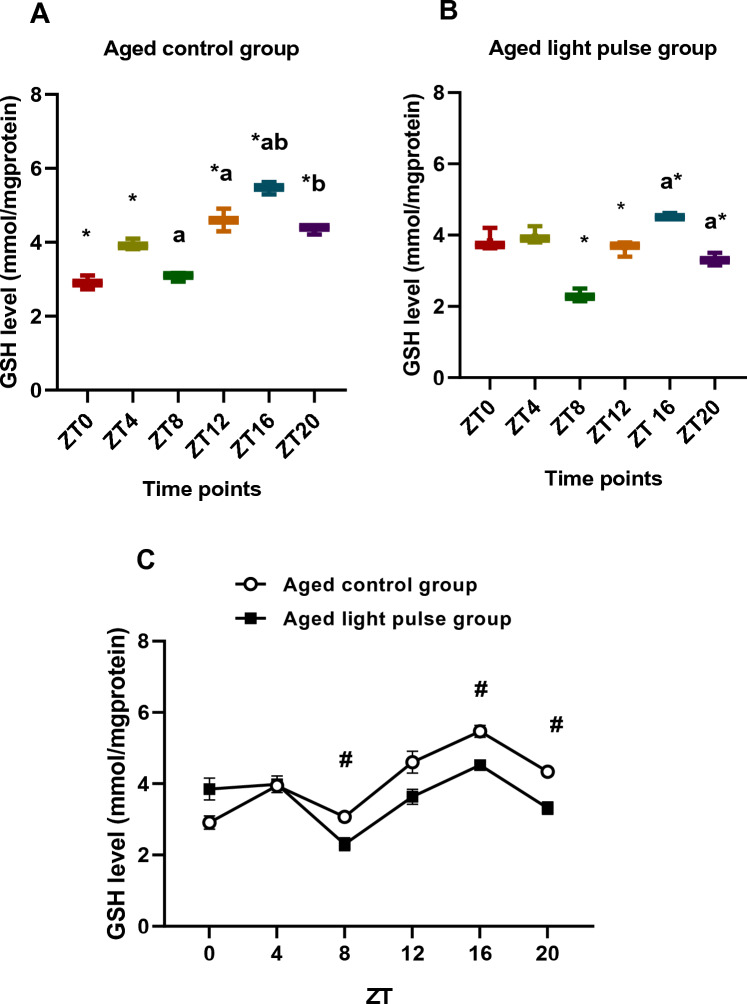
**A**, **B** Box and whisker plot showing the distribution of GSH levels in the liver tissue among different time points in aged control group (**A**) and aged light pulse group (**B**). In the aged control group (**A**), * significance at ZT 4, ZT12, ZT16, and ZT20 vs ZT 0; ^a^ significance at ZT12, ZT16, and ZT20 vs ZT8; ^b^ significance at ZT20and ZT16 vs ZT4. In the aged light pulse group (**B**), *significance at ZT12, ZT16, and ZT20 vs ZT8; ^a^ significance between ZT16 and ZT20. **C** A daily profile of GSH levels in the liver tissue of both aged control group and aged light pulse group. Data represented as mean ± SE. ^#^ Significance at ZT8, ZT16, and ZT20 between the 2 groups

Levels of GSH in aged control rats recorded great variations during all-time points represented by box and whisker plots. The significant maximum value (*p < *0.05) was at ZT16 compared to all time points (Fig. 5A). In the same way, rats exposed to a 30-min light pulse showed differences during all-time points, which was indicated by box and whisker plots. Significant values (*p < *0.05) of GSH level were at ZT12, ZT16, and ZT20 compared with ZT8. Moreover, there was a significant difference between ZT16 and ZT20 (Fig. 5B). The current data clarifies that GSH increased at night time. Two-way ANOVA showed a highly significant effect of time (*p < *0.0001), a significant effect of group (*p < *0.0001), and a significant interaction effect between the two groups (*p < *0.0001). Levels of GSH were diminished significantly (*p < *0.05) in the aged-light pulse group at ZT8, ZT16, and ZT20 compared to that of the corresponding time points in aged control rats (Fig. 5C).

## Discussion

Light is the powerful entraining factor used to combat disruptions of the circadian clock and improve disturbances caused by shift work [20]. Moreover, our previous study clarified the significance of light pulse in ameliorating circadian rhythm disturbances associated with the aging process. Light pulse at late night (ZT22) attenuated age-related neurodegeneration in rats [13]. Circadian rhythm disturbances during aging are characterized by a diminish in the clock gene expression in the SCN and peripheral organs. The downregulation of the *Per2* clock gene is implicated in signs of aging [21].

*Per2* mRNA is expressed in the liver of adult rats with a peak at night, during its activity phase, and a reduction within the light period [22]. In the present study, we noticed the same circadian rhythm in *Per2* mRNA in aged rat liver with a peak at the dark phase and a trough during the light phase. The *Per2* gene is the clock component that responds to external signals such as light [23]. Herein, the treatment with 30 min light pulse late at night, at ZT 22, abolished *Per2* circadian rhythm and downregulated its gene expression in the liver. Similarly, Fonken and Nelson, [15] reported that chronic dim light at night suppressed the *Per*1 and *Per2* gene expressions in the liver but attenuated their rhythm in the SCN around the light/dark transition. In another study, dim light at night did not influence the rhythm of clock gene expression in the SCN or liver of zebra finch [24]. Furthermore, the application of light pulse late at night, CT 19, induced *Per2* mRNA in the SCN of aged hamsters [25]. The first explanation of *Per2* mRNA arrhythmicity in the liver, of the present study, may result from using low-intensity light (100 lx). Hamada et al. [26] found that only high-intensity light (1000 lx, but not 100 lx), induced *Per2* mRNA expression in the olfactory bulb of mice. On the contrary, it failed to induce *Per2* gene expression in the parietal cortex [26]. Second, the peripheral clocks shift more slowly and in different ways than in SCN [15]. Third, the lack of circadian rhythm in the peripheral organs does not mean the cells lose their rhythmicity, but it may be due to intercellular desynchronization [27].

In the present study, gene expression of *Bcl2*, the anti-apoptotic member of the Bcl-2 family, was diminished in the liver of aged rats after applying a light pulse late at night. Nahata et al. [28] brought to light the inverse relation between the autophagy process in the liver of aged mice and *Bcl2* gene expression. *Bcl2* mRNA increases in the liver with aging and is associated with diminished autophagy capacity of it. The autophagy process is essential for hepatic cells to perform metabolic functions such as gluconeogenesis and β-oxidation. Thus, exposure to a light pulse in this study may enhance the autophagy process in the liver through the downregulation of the *Bcl2* gene.

Aging is associated with increased proinflammatory processes and tissue remodeling without a compensating stimulation of antioxidant response genes. Therefore, shifting the balance toward a pro-oxidant and proinflammatory state “inflamm-aging” [29]. Moreover, Dato et al. [12] clarified that the continuous reduction of free radicals by antioxidants is harmful to healthy aging. They explain the value of ROS production during aging by its ability to generate a hormetic mechanism. Mitochondrial hormesis considers ROS as a signal produced in mitochondria and transported to other cellular organelles in response to pathophysiological stimuli. ROS signals activate specific cascades resulting in an improvement in the antioxidant state, delay age-related changes and promote longevity.

Hepatic aging is associated with the accumulation of lipid peroxidation products and oxidized/carbonylated proteins in rhesus monkeys [30]. Light pulse late at night, in the current study, enhanced the reduction of the lipid peroxidation process, represented as MDA decrement, in the hepatic cells of the aged rat. In addition, it was not capable of reducing NO or induce the production of the antioxidant, GSH. Mohamed et al. [31] found a decrease in ROS, MDA, NO, NF-κB p65, TGF-β1, pro-inflammatory mediators, and increased antioxidant defenses in the liver of rats after performing low-level laser therapy. On the contrary, continuous blue light application, but not a green light, elevated liver ROS, MDA and reduced the activity of GSH Peroxidase resulting in inadequate antioxidant capacity in the liver. Moreover, it disrupted the expression of clock genes in the hypothalamus, elevated the plasma corticosterone level, and increased glutathione reductase synthesis and transport which in turn led to oxidative stress in the liver [32].

Critical oxidative stress induces casein kinase II (CKII) which in turn activates the circadian clock genes, Bmal1and Clock, that results in the upregulation of the *Nrf2* gene. Nrf2 protein then activates antioxidant genes that encode glutathione s-transferase-alpha2 enzyme [33]. Upon encountering a xenobiotic, the glutathione s-transferase enzyme catalyzes the conjugation of glutathione with the xenobiotic [34]. This reaction may explain why GSH was decreased in this study**.**

The activation of CKII with ROS leads to the phosphorylation of *Bmal1* and heat shock factor, which in turn induces the transactivation of *per2* and other genes so resetting the clock [33]. In response to oxidative stress, *Per2* regulates cell death by modulating the redox state of a cell. Additionally, the redox state of a cell plays a vital role in the regulation of clock gene transcription via the NAD-dependent enzyme SIRT1 [35]. Overexpression of *Per2* in the carcinoma cell line leads to a downregulation of the *Bcl2* gene and increased apoptosis [36]. Furthermore, Magnone et al. [37] revealed an increase in the resistance to oxidative stress in the cell line of mice with a mutation in the *Per2* gene. Taken together, the *Per2* gene optimizes the balance between cell death and cell survival and affects the cellular response to oxidative stress in a manner that influences cancer development and the aging process [37].

## Conclusion

In conclusion, the light pulse at late night abolished the rhythm of *Per2* mRNA and downregulated its expression in aged rat liver. Additionally, *Bcl2* gene expression was downregulated, which might induce apoptosis in the aged liver. Furthermore, it might reduce oxidative stress partially by decreasing MDA levels in hepatic cells.
